# Morphological Study of First Instar Elephant Stomach Bot Fly Larvae (Oestridae: Gasterophilinae: *Cobboldia elephantis*)

**DOI:** 10.3390/insects16070733

**Published:** 2025-07-18

**Authors:** Xingkun Yang, Zhuowei An, Chaoyong Xiong, Shenming Tan, Mingwei Bao, Fangyi Zhou, Meiqin Liu, Liping Yan, Dong Zhang, Thomas Pape

**Affiliations:** 1 School of Ecology and Nature Conservation, Beijing Forestry University, Qinghua East Road 35, Beijing 100083, China; xingkun_yang@bjfu.edu.cn (X.Y.); anlinsener@163.com (Z.A.); yanlp@bjfu.edu.cn (L.Y.); 2 Asian Elephant Protection Management Center of Xishuangbanna Dai Autonomous Prefecture, Jinghong 666100, China; 18988133909@163.com; 3 Yunnan Xishuangbanna National Nature Reserve Management Bureau, Jinghong 666100, China; tsm119567@163.com; 4 Wild Elephant Valley Asian Elephants Provenance Breeding and Rescuecenter of Xishuangbanna, Jinghong 666100, China; baomingwei-11@163.com (M.B.); zhoufangyi@mail.bnu.edu.cn (F.Z.); 5 Analysis and Testing Center, Beijing Forestry University, Beijing 100083, China; lpqmq@bjfu.edu.cn; 6 Natural History Museum Denmark, Universitetsparken 15, 2100 Copenhagen, Denmark; tpape@snm.ku.dk

**Keywords:** *Cobboldia elephantis*, confocal laser scanning microscopy, evolutionary adapation, morphological comparison, scanning electron microscopy, ultrastructure

## Abstract

*Cobboldia elephantis* (Oestridae: Gasterophilinae) is an obligate parasite of the alimentary tract of the Asian elephant, causing gastric myiasis. Current knowledge of its first instar larval morphology has been limited to observations under light microscopy, significantly constraining our understanding of morphological evolution within the genus *Cobboldia* and the broader subfamily Gasterophilinae. In this study, we provided ultrastructural and three-dimensional characterizations of *C. elephantis* using scanning electron microscopy (SEM) and confocal laser scanning microscopy (CLSM) based on newly hatched first instar larvae. Features of the first instar larva of *C. elephantis*, which are either unique or shared with *C. loxodontis* Brauer, 1896, include (i) three pairs of mouthhooks, (ii) posterior spiracles bearing peristigmatic tufts, (iii) anal division with two spine-clusters and a row of elongated spines, and (iv) a planed, button-like sensilla and a sensillum placodeum. We also compared the first instar larval morphology of *C. elephantis* with that of *Cobboldia loxodontis*, *Gyrostigma rhinocerontis* (Owen, 1830), *Gasterophilus pecorum* (Fabricius, 1794), *Portschinskia magnifica* Pleske, 1926, and *Oestrus ovis* Linnaeus, 1758. Species of Gasterophilinae share several unique features of the first instar larva, including (i) an anal division composed of three subdivisions, (ii) spiracular slits on the posterior spiracles, and (iii) conserved positioning of thoracic sensilla. These findings fill a key gap in our knowledge of *C. elephantis* larval morphology and suggest that these distinctive structures play a role in adaptation to its parasitic lifestyle.

## Simple Summary

In the obligate parasitic Oestridae family, morphological studies of larvae are relatively scarce, limiting our understanding of their parasitic lifestyles. This study employed scanning electron microscopy and confocal laser scanning microscopy to document and analyze the three-dimensional configurations and ultrastructures of first instar larvae of *Cobboldia elephantis* (Steel, 1878), providing detailed descriptions of their mouthhooks, spine distribution, sensory receptor types, and posterior spiracles. Notably, this species is the first in Oestridae to exhibit peristigmatic tufts. By comparing with species of the three other genera of stomach Gasterophilinae, we confirmed their phylogenetic relationships. This research fills several gaps in morphological studies of first instar larvae of *C. elephantis* and offers new insights into the adaptive evolution of stomach bot flies with their hosts.

## 1. Introduction

*Cobboldia elephantis* (Steel, 1878) (Oestridae: Gasterophilinae) belongs to the obligate parasitic subfamily Gasterophilinae, whose species complete their larval development within the alimentary tract of large, non-ruminant mammals, causing gastric myiasis [1,2,3]. Numerous reports from regions such as Andhra Pradesh and Tamil Nadu, India, as well as Bago Region, Myanmar, have documented severe infestations of *C. elephantis* larvae in both wild and captive Asian elephants, often discovered during necropsies [4,5,6,7]. In China, larvae were first identified in the feces of Asian elephants [8], highlighting the potential threat this parasitic species poses to the conservation of Asian elephant populations across their range.

Most of the hosts of Gasterophilinae—including equids, elephants, and rhinoceroses—are generally threatened or endangered [1,9]. Consequently, many species of Gasterophilinae are rare or endangered. Notably, the adaptive radiation of Gasterophilinae is thought to have occurred within a relatively short evolutionary timespan [10], making this group a valuable model for studying host–parasite coevolution. Despite their evolutionary and ecological significance, our understanding of the developmental and adaptive traits of Gasterophilinae remains limited.

Gasterophilinae comprise three genera: *Cobboldia* Brauer, *Gasterophilus* Leach, and *Gyrostigma* Townsend. While the micro- and ultrastructural features of *Gasterophilus* have been well documented across all developmental stages, and partially so in *Gyrostigma* [11,12,13,14,15], knowledge of *Cobboldia* larval morphology—especially in the first instar—remains sparse. This is primarily due to difficulties of collecting samples when eggs are glued to the base of host elephants’ tusks and require strict hatching conditions within certain temperature and moisture ranges, and because of the brief first instar duration. Patton (1922) first observed *C. elephantis* first instar larvae by hatching egg clusters under laboratory conditions [16]. The next morphological account of this species only appeared more than 100 years later, when this species was first reported in China [8]. However, the ultrastructural features and the potential coevolutionary adaptations remained largely unexplored.

Light microscopy (LM) has long been employed in larval studies but is limited in its ability to resolve fine morphological details [17]. Scanning electron microscopy (SEM) offers high-resolution imaging of surface structures [18], while confocal laser scanning microscopy (CLSM) provides complementary advantages, such as non-destructive visualization of internal features using the natural autofluorescence of insect tissues [19]. CLSM enables high-fidelity, three-dimensional reconstruction of delicate structures—especially valuable for studying the minute, fragile bodies of first instar larvae [17,18,19].

In this study, we described the ultrastructural and three-dimensional morphology of *C. elephantis* first instar larvae using both SEM and CLSM. We also conducted a comparative morphological analysis with the first instar larvae of *Cobboldia loxodontis* Brauer, 1896 [20], *Gyrostigma rhinocerontis* (Owen, 1830), *Gasterophilus pecorum* (Fabricius, 1794) [21], *Portschinskia magnifica* Pleske, 1926 [22], and *Oestrus ovis* Linnaeus, 1758 [23]. Our goals were to establish reliable diagnostic character states for species identification and to provide new insights into the phylogenetic relationships and potential adaptations of this poorly understood taxon.

## 2. Materials and Methods

### 2.1. Sample Collection

*Cobboldia elephantis* deposits its eggs in clusters consisting of rows of 20 to 40 eggs glued to the base of elephant tusks [8,20]. We collected the egg clusters from habituated Asian elephant hosts at the Wild Elephant Valley Asian Elephant Provenance Breeding and Rescue Center in Xishuangbanna, Yunnan, China. During routine cleaning and care activities for Asian elephants, the sixth author of this study discovered and collected egg clusters by hand at the base of the elephants’ tusks. The collection process was conducted in two phases: (1) On 3 April 2024, a total of 132 egg clusters were collected from six female (age range: 5–39 years) and three male (age range: 26–35 years) Asian elephants; (2) on 11 April 2025, 89 egg clusters were collected from a seven-year-old male Asian elephant. The collected egg clusters were placed in 50 mL centrifuge tubes with perforations for ventilation and subsequently transported to Beijing Forestry University, Beijing, China. The first and second authors of this study conducted the incubation of the egg clusters under laboratory conditions [8].

Trials showed that egg clusters containing mature first instar larvae of *C. elephantis* require a highly humid environment for hatching [8]. To induce hatching, egg clusters were placed in a Petri dish, and deionized water was added to ensure the water level completely submerged the egg clusters, keeping them fully immersed and moist. A constant-temperature heating platform was placed beneath the dish to maintain a steady temperature of 35 °C. Approximately 1–2 min later, the larvae began to push open the operculum using their pseudocephalon and thorax. When the operculum had been pushed up to create a distinct gap, the larvae rapidly wriggled their bodies to emerge from the egg. Subsequently, the larvae were killed using 95 °C hot water, preserved in 75% ethanol, and stored at −20 °C. A total of 63 first instar larvae were successfully obtained.

### 2.2. Sample Observation, Imaging, and Terminology

For SEM, five larval specimens were processed as follows: two for dorsal view imaging, two for ventral view imaging, and one for frontal view imaging of the pseudocephalon and posterior spiracles. The larval specimens were initially cleaned via ultrasonic treatment (60 Hz 10 min) in a cleaning solution (Kun Shan Ultrasonic Instruments Co., Ltd., Kunshan, China, KQ5200E), followed by rinsing thrice with room temperature PBS buffer (Solarbio, Beijing, China) to remove residues. Dehydration was performed using a gradient ethanol immersion method (70%, 80%, 90%, 95%, and 100% ethanol, 30 min per gradient). To obtain a frontal view of the pseudocephalon and posterior spiracles, one larva was sectioned at the second thoracic segment and the sixth abdominal segment using a thin blade, retaining only the parts containing the pseudocephalon and posterior spiracles. All specimens were adjusted to appropriate angles and affixed to aluminum stubs using conductive adhesive, then dried in a desiccator for at least 24 h. After air-drying, the specimens were sputter-coated with gold using an ion sputter coater and examined under a field-emission scanning electron microscope (SU8010, Hitachi, Tokyo, Japan) [21].

For CLSM, three first instar larval specimens were selected for dorsal imaging and three for ventral imaging. The larval specimens were initially rinsed with pre-cooled PBS buffer (pH 7.0) to remove surface contaminants, followed by tissue clearing in 10% KOH solution (pH 12.6) for 24 h at room temperature. The tissue clearing process effectively removed pigments and lipids from the larval specimens, significantly enhancing optical transparency and allowing clear visualization of internal structures under the confocal microscope. After neutralization with PBS buffer (pH 7.0, verified by pH test strips), the specimens were carefully transferred to concave slides using a micropipette and mounted in 50% glycerol aqueous solution. Fluorescence imaging was performed using a Leica TCS SP8 confocal system equipped with triple-laser excitation (405 nm, 488 nm, 633 nm), with photomultiplier tube (PMT) detectors configured to capture autofluorescence signals in three spectral ranges: 410–468 nm (blue), 508–534 nm (green), and 644–759 nm (red). Fluorescence imaging further revealed the fine details of the larval cuticle, cephaloskeleton, and internal cuticular structures, providing high-resolution images for subsequent morphological analysis [17,20].

Terminology for larval morphology follows recent work on bot fly immatures [12,21].

### 2.3. Morphological Comparison

To undertake a comparative investigation of the morphological structure of *C. elephantis*, we selected the congeneric *Cobboldia loxodontis* [20] and species for which the first instar larva has been imaged using confocal laser scanning microscopy (CLSM) or scanning electron microscopy (SEM), including representatives for the remaining genera of Gasterophilinae *Gyrostigma rhinocerontis* and *Gasterophilus pecorum* [21], as well as *Portschinskia magnifica* from Hypodermatinae [22], and *Oestrus ovis* from Oestrinae [23].

## 3. Results

### 3.1. Morphological Characteristics of First Instar Larvae in Cobboldia elephantis

**Figure 1:**
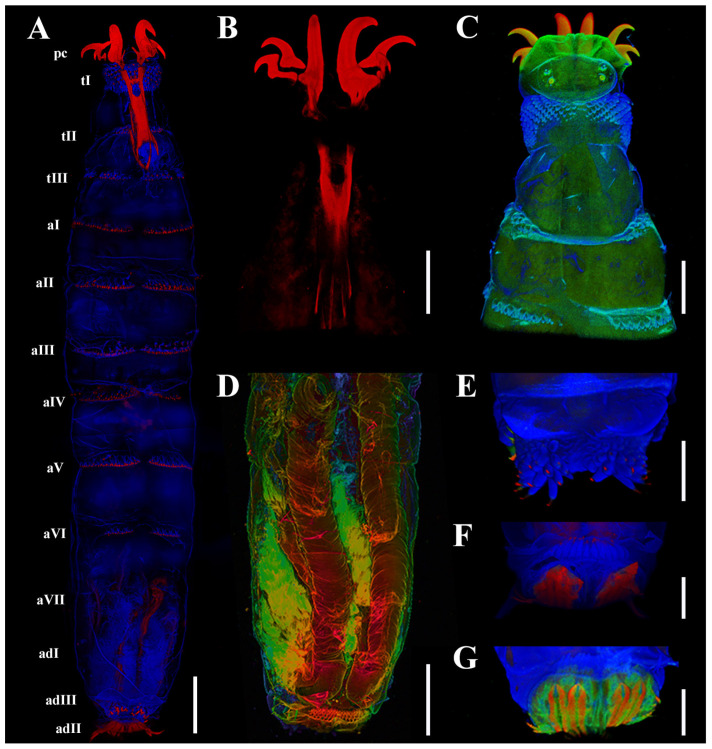
*Cobboldia elephantis* (Steel, 1878) first instar larva, confocal laser scanning microscopy details, 410–468 nm (blue), 508–534 nm (green), and 644–759 nm (red). (**A**) Habitus, ventral view. (**B**) Pseudocephalon and thoracic segments, dorsal view. (**C**) Pseudocephalon and thoracic segments, dorsal view. (**D**) Tracheal structures. (**E**) Spines of subdivision II of anal division. (**F**) Spiracular slits. (**G**) Spiracular plate. Scale bars: (**A**,**D**) 200 μm, (**B**,**C**,**E**–**G**) 100 μm. Abbreviations: aI–aVII, abdominal segments I–VII; adI–adIII, subdivisions I–III of anal division; pc, pseudocephalon; tI–tIII, thoracic segments I–III.

**Figure 2:**
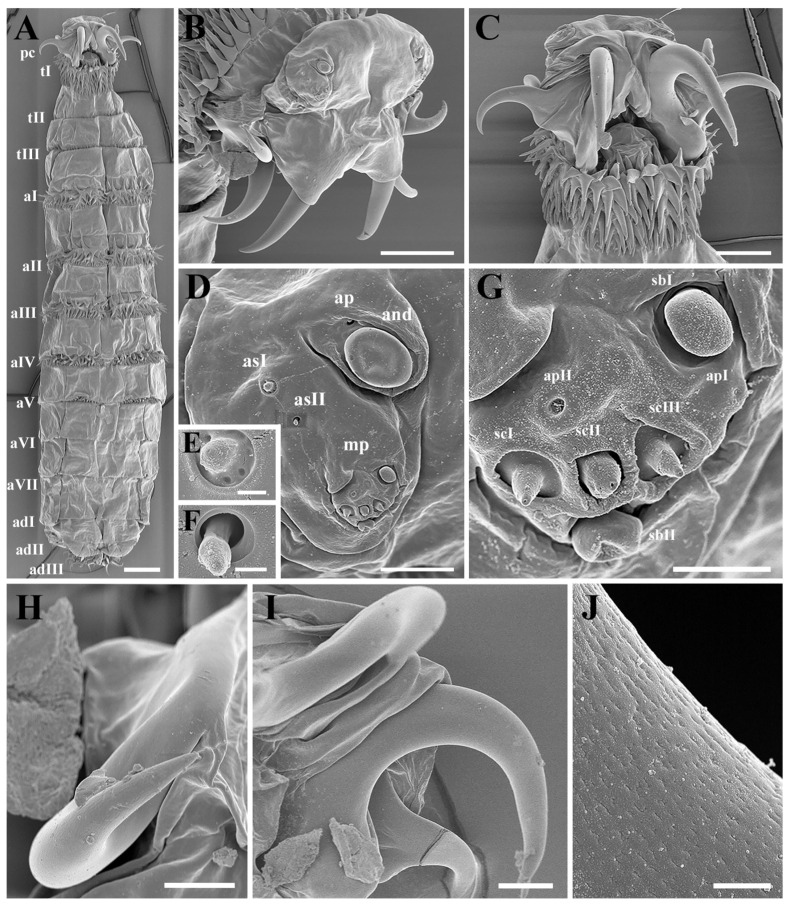
*Cobboldia elephantis* (Steel, 1878) first instar larva, scanning electron microscopy details of habitus, pseudocephalon, and mouthhooks. (**A**) Habitus, ventral view. (**B**) Pseudocephalon, latero-dorsal view. (**C**) Pseudocephalon, ventral view. (**D**) Antennomaxillary sensory complex. (**E**,**F**) Additional sensillum I and II. (**G**) Maxillary palp. (**H**) Accessory mouthhook. (**I**) Primary mouthhook. (**J**) Cuticular surface from the middle part of the primary mouthhook. Scale bars: (**A**) 100 μm, (**B**,**C**) 50 μm, (**D**,**E**) 9 μm, (**F**) 600 nm, (**G**) 2.5 μm, (**H**) 10 μm, (**I**) 25 μm, (**J**) 1.5 μm. Abbreviations: adI–adIII, subdivisions I, II, and III of the anal division; aI–aVII, abdominal segments I–VII; and, antennal dome; asI–II, additional sensillum I–II; apI–II, additional pit I–II; mp, maxillary palp; pc, pseudocephalon; sbI–II, sensillum basiconicum I–II; scI–III, sensillum coeloconicum I–III; tI–tIII, thoracic segments I–III.

*Habitus*: The first larval instar of *C. elephantis* has a fusiform body, a bilobed pseudocephalon (pc), three thoracic segments (tI–tIII), seven abdominal segments (aI–aVII), and an anal division (ad) composed of three subdivisions (adI–adIII) (Figure 1A,E,F and Figure 2A). Each body segment is equipped with a number of shallow pits distributed linearly, dorsally, and ventrally, as well as spinose bands anteriorly, except for the last two subdivisions of the anal division (adII–III).

*Cephaloskeleton*: A pair of strong primary mouthhooks each has a pair of smaller accessory mouthhooks connected to the base of the primary mouthhook (Figure 1B), and all mouthhooks reach out of the oral cavity for a considerable distance (Figure 2A–C). All mouthhooks are well developed, strongly sclerotized, and sickle-shaped, with a stout base and sharp tip, and short, irregular grooves presented on the surface (Figure 2J). The primary mouthhook is directed ventrally, and the two accessory mouthhooks are bent ventro-laterally and laterally or even latero-dorsally (Figure 2B,C,H,I). A labrum is absent (Figure 1A,B).

*Pseudocephalon*: The anterior end possesses a pair of antennomaxillary sensory complexes, each consisting of an antennal dome (and), a maxillary palpus (mp), two additional sensilla (as), and a set of additional pits (ap) (Figure 2B,D,G). The antennal dome is circular, flattened, and slightly elevated from the surrounding cuticle (Figure 2D), and the additional sensillum I and II are situated dorsolaterally (Figure 2E,F). The maxillary palpus consists of two equal-sized sensilla basiconica (sbI–sbII), three sensilla coeloconica (scI–scIII), and two additional pits (apI–apII), with the bases of the sensilla coeloconica sunken in cavities.

**Figure 3:**
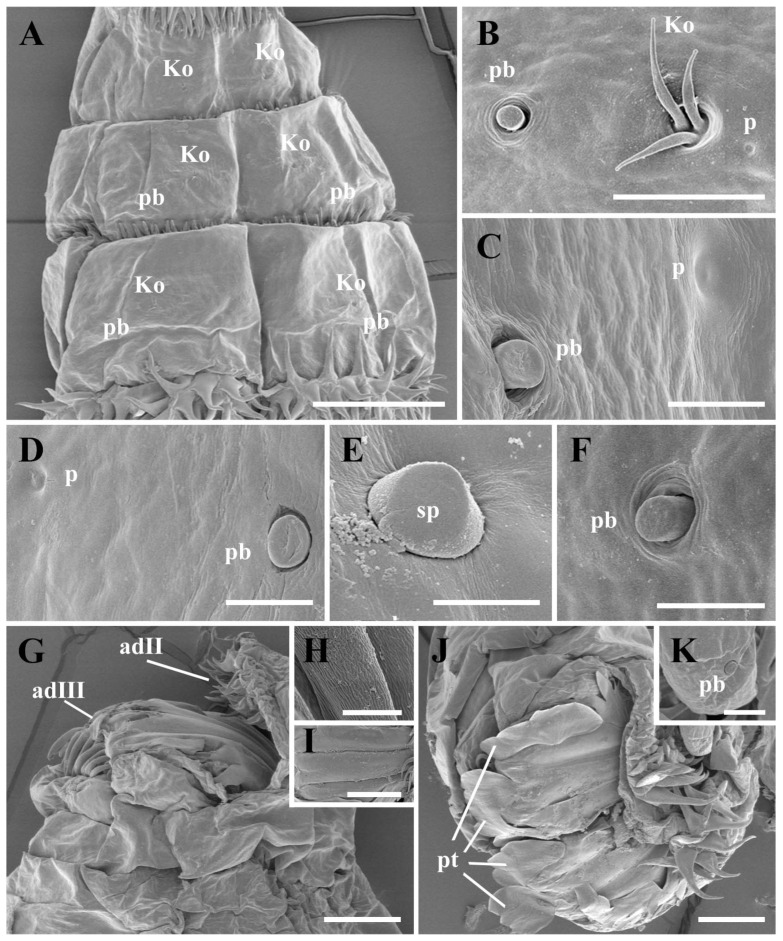
*Cobboldia elephantis* (Steel, 1878) first instar larva, scanning electron microscopy details of thoracic (**A**–**C**) and abdominal (**D**–**F**) segments and anal division (**G**–**K**). (**A**) Three thoracic segments, ventral view, showing arrangement of Keilin’s organs and trichoid sensilla. (**B**) Thoracic segment III of Keilin’s organs and a small hole next to it, planed button, ventral view. (**C**) Abdominal segment II of planed button and pit, dorsal view. (**D**) Abdominal segment I of planed button and pit, dorsal view. (**E**) Abdominal segment IV of sensillum placodeum and pit. (**F**) Planed button on abdominal segment IV. (**G**) Anal division, lateral view. (**H**) Enlarged view of anal division spine. (**I**) Enlarged view of spiracular slits. (**J**) Anal division, posterior view. (**K**) Enlarged view of planed button in protuberance. Scale bars: (**A**) 100 μm, (**B**) 10 μm, (**C**,**D**) 5 μm, (**E**) 2.5 μm, (**F**) 5 nm, (**G**) 50 μm, (**H**) 4 μm, (**I**) 5 μm, (**J**) 50 μm, (**K**) 25 μm. Abbreviations: adII–adIII, subdivisions II and III of anal division; Ko, Keilin’s organ; p, pit; pb, planed button; pt, peristigmatic tuft; sp, sensillum placodeum.

*Thorax*: The first thoracic segment (tI) is encircled with rows of recurved sharp spines, with seven to nine rows of spines dorsally and seven to eight rows ventrally, exhibiting an alternating distribution pattern. The second and third thoracic segments (tII–tIII) have two to three rows of spines dorsally and ventrally (Figure 1C). Each thoracic segment present discal ventrolateral spines. The Keilin’s organs (Kos) are paired structures, consisting of three trichoid sensilla, situated ventrally on tI–tIII, and shallow pits were noted on the inside surface. On tII–tIII, each Keilin’s organ is accompanied laterally by a button-like sensillum basiconicum, where the peg is flattened and more or less level with the surrounding cuticle, henceforth referred to as a planed button (Pb) (Figure 3A). Additionally, planed buttons are present scattered dorsally on tI–tIII, each with a shallow pit (Figure 3C).

*Abdomen*: The first to sixth abdominal segments (aI–aVI) are each encircled by two to four rows of regularly arranged spines that gradually decrease in size towards the posterior end of the body (Figure 1A and Figure 2A). The first three thoracic segments present discal ventrolateral spines. Sensilla placodea (sp) were found on the dorsal surface of all segments except the fourth abdominal segment, while planed buttons were found on the dorsal surface of all segments, situated in shallow pits (Figure 3D–F).

*Anal division*: The anterior part of each subdivision has no spine distribution, and two clusters of spines grow at the end of second subdivision (adII) (Figure 1E and Figure 3G,J). The posterior spiracles (ps) are not protruding from the body surface, with each spiracle presenting two spiracular slits and three peristigmatic tufts (pt), each of which has numerous short branches (Figure 3G,I,J) and longer spines ventrally, measuring 4–5 times the length of the spines in the second subdivision (Figure 3H). Moreover, the second subdivision displays a shallow median concavity dorsally and ventrally with two papillae, with one additional tubercle laterally on either side, all with a planed button apically (Figure 3K).

The most important morphological differences of first instar larvae among six species of Oestridae are shown in Table 1.

### 3.2. Autofluorescence Spectral Range and Intensity

Fluorescence imaging revealed distinct tissue-specific emission patterns under different excitation wavelengths [21]. The 405 nm laser primarily activated fluorescence signals from the larval integument and tracheal network, while 488 nm excitation showed stronger affinity for muscle tissue visualization (Figure 1A,C,D). Sclerotized structures such as cephaloskeletal components, along with select regions of major tracheal branches, only produced detectable fluorescence when stimulated by 633 nm irradiation (Figure 1A,C,D). Spectral analysis further identified regional variations in spine fluorescence characteristics, with the distal extremities of all spines exhibiting distinct red fluorescence (Figure 1A,E,F).

## 4. Discussion

### 4.1. Morphological Comparison of Cobboldia elephantis with Related Species

A comparative analysis was conducted on several morphological structures of phylogenetic significance [2].

(i) Cephaloskeleton: Most remarkably, the genus *Cobboldia* possesses three pairs of well-developed mouthhooks, with the configuration in *C. elephantis* being very similar to that in *C. loxodontis* [20]. The labrum is absent in both species of *Cobboldia* as well as in *O. ovis* and all other species of subfamily Oestrinae, which may indicate that the first instar *Cobboldia*, like the nasal bot flies, does not have an early subdermal migration. (ii) Pseudocephalon: The antennomaxillary sensory complex in Gasterophilinae exhibits a similar composition and is more complex than that of *P. magnifica*, though *C. elephantis* has fewer additional sensilla. (iii) Thoracic Structure: The distribution of Keilin’s organs is consistent within Gasterophilinae, while *P. magnifica* lacks this structure. *Portschinskia magnifica* exhibits a unique feature: a cap-like sclerotization dorsally on the second thoracic segment. (iv) Anal Division: In Gasterophilinae, it consists of three subdivisions, with eight small coeloconic sensilla distributed on the second subdivision in *Ga. pecorum* and *Gy. rhinocerontis*, and only their posterior spiracles protrude from the body. In *C. elephantis*, six symmetrically arranged papillae are present in the second subdivision, each featuring an apical planed button. *Oestrus ovis* possesses trichoid sensilla and coeloconica sensilla. Notably, the spiracular surface is encircled by peristigmatic tufts, a structure found throughout Cyclorrhapha but here recorded for the first time in Oestridae [24,25,26]. The posterior spiracles in Gasterophilinae open via spiracular slits, while *P. magnifica* and *O. ovis* open through spiracular plates. (v) Spines: The spines of the three Gasterophilinae species are hook-shaped, but the number of rows varies significantly. *Portschinskia magnifica* primarily exhibits scale-like spines, whereas *O. ovis* has thin scale-like spines with 2–3 small teeth at the tips. Except for *P. magnifica*, all other species possess discal ventrolateral spines. Regarding the spines surrounding the posterior spiracles, only *C. elephantis* within Gasterophilinae exhibits a single row of elongated spines, distinct from the well-developed, hook-shaped spines of *O. ovis*. CLSM reveals that only the tips of these spines emit red fluorescence in *C. elephantis*. (vi) Sensilla: Only *C. elephantis* exhibits planed buttons and sensilla placodea distributed across its body surface (Table 1).

*Cobboldia elephantis* displays a set of character states that together characterize the first instar larvae of Gasterophilinae [21]: (i) anal division composed of three subdivisions; (ii) posterior spiracles open on the third subdivision (adIII), with two slit-shaped spiracular slits on each side; (iii) hook-shaped spines uniformly positioned along the anterior margin of each body segment, with their apices directed posteriorly; and (iv) ventral surface of all three thoracic segments characterized by a pair of Keilin’s organs, with each organ flanked laterally by a sensillum on both the second and third thoracic segments. Additionally, for *Cobboldia*, the presence of three pairs of mouthhooks is a distinctive and unique feature (Table 1).

Previous morphological and molecular studies have demonstrated that the subfamily Gasterophilinae constitutes a monophyletic group, with the genera *Gasterophilus* and *Gyrostigma* forming a sister-group relationship [10,27,28]. The present comparative morphological analysis of first instar larvae provides further support for these findings.

**Table 1:** Morphological differences of first instar larvae among six species of Oestridae. Unknown or inapplicable character states given as “–”.

Morphological Structure	*Cobboldia elephantis*	*Cobboldia loxodontis* [20]	*Gyrostigma rhinocerontis* [21]	*Gasterophilus pecorum* [21]	*Portschinskia magnifica* [22]	*Oestrus ovis* [23]
**General body, shape**: (0) fusiform; (1) flattened dorsoventrally	0	0	0	0	0	1
**Mouthhooks, accessory pair laterally to each primary mouthhook**: (0) absent; (1) present	1	1	0	0	0	0
**Mouthhooks, shape**: (0) distinctly curved, hook-shaped; (1) almost straight, with a small hook apically; (2) almost straight, triangular tip with small ventral barb; (3) slightly curved, gradually tapering	0	0	1	1	2	3
**Mouthhooks, orientation**: (0) dorsally; (1) ventrally; (2) ventro-laterally	1	–	0	0	2	2
**Labrum, presence**: (0) absent; (1) present	0	0	1	1	1	0
**Labrum, length**: (1) long; (2) short	–	–	1	1	1	–
**Labrum, shape**: (0) sword-like with a sharp tip; (1) sword-shaped with a slightly obtuse tip; (2) triangulum minor	–	–	0	1	2	–
**Denticles around oral opening**: (0) absent; (1) present	0	–	1	1	0	0
**Antennal dome, shape**: (0) peg-like; (1) flat, button-like	1	1	1	1	0	0
**Antennomaxillary sensory complex, additional sensilla**: (0) two; (1) three; (2) four	0	–	1	2	0	–
**Antennomaxillary sensory complex, number of sensilla coeloconica**: (0) three; (1) absent	0	–	0	0	1	–
**Dorsal sclerotisation on first thoracic segment**: (0) absent; (1) present	0	–	0	0	1	–
**Spines, distribution**: (0) to abdominal segment 7; (1) to abdominal segment 6 and anal division; (2) to abdominal segment 4 and anal division	1	2	0	0	1	1
**Discal ventrolateral spines**: (0) absent; (1) present	1	1	1	1	0	1
**Spines around the posterior spiracular plate**: (0) absent; (1) a row of longer spines; (2) a pair of thick spines and a spinose band confined to the cuticle; (3) a row of large, well-developed spines	1	1	0	0	2	3
**Thoracic and abdominal spines, shape**: (0) hook-shaped; (1) the first row of spines in the band of spines below pseudocephalon are hooked, while the others are scaly; (2) thin scale-like spines, tips with 2–3 small teeth	0	–	0	0	1	2
**Keilin’s organs**: (0) absent; (1) present	1	–	1	–	1	1
**Accompanying sensilla of Keilin’s organs**: (0) planed button; (1) trichoid sensilla; (2) coeloconic sensilla	0	–	1	1	3	2
**Posterior spiracles**: (0) non-extended (1) protruding	0	0	1	1	0	0
**Peristigmatic tufts**: (0) absent; (1) present	1	–	0	0	0	0
**Posterior spiracular opening**: (0) spiracular slits; (1) spiracular plates	0	–	0	0	1	1
**Sensilla on anal division**: (0) planed button; (1) sensilla coeloconica; (2) trichoid sensilla and sensilla coeloconica	0	–	1	1	1	2
**Red fluorescence of spines**: (0) excited only at tip; (1) excited overall	0	–	1	1	1	–
**Red fluorescence of posterior tracheal trunks**: (0) only in anal division; (1) extending anterior to anal division	1	–	1	1	0	–

### 4.2. Inferences on the Functional Adaptations of Morphological Structures

Species of the family Oestridae are obligate parasites within mammals, and their morphological structures have undergone adaptive radiation to better perform related functions, thereby giving rise to unique parasitic strategies. In this process, the morphology of the mouthhooks in first instar larvae of Oestridae has diversified significantly, exhibiting corresponding adaptations to parasitic life [2,3,29,30]. The larvae of the subfamily Hypodermatinae possess relatively small, crescent-shaped, and strongly curved mouthhooks [31]. In contrast, the larvae of the subfamilies Oestrinae and Cuterebrinae have prominent mouthhooks that are slightly curved, gradually tapering, and oriented latero-ventrally or ventrally [22,32]. Most strikingly, for the subfamily Gasterophilinae, the larvae exhibit elongate, heavily sclerotized mouthhooks with curved tips. While the first instar larvae of species of the genera *Gasterophilus* and *Gyrostigma* have elongated mouthhooks and a distinct labrum [21], those of the genus *Cobboldia* show no trace of a labrum, and in addition to the pair of distinctive mouthhooks, they have two pairs of accessory mouthhooks oriented dorsally and posteriorly, while the primary mouthhooks are oriented ventrally. This significant difference suggests a highly specialised migration strategy of *Cobboldia* first instar larvae within the host, potentially relying more on the coordinated extension and retraction of the mouthhooks, and with the accessory mouthhooks as additional support when migrating along the mucosa of the mouth or tongue of the host elephant. The homology of the accessory mouthhooks needs further study, but they appear to be part of the cephaloskeleton and may as such be comparable, if not homologous to the dental and accessory oral sclerites seen in several Muscidae [33].

Gasterophilinae larvae parasitize the host’s digestive tract, which requires efficient gas exchange capability to survive. CLSM shows that the posterior tracheal trunks of *Portschinskia magnifica* first instar larvae induce the emission of red fluorescence from the part found in the anal division [29], while first instar larvae of Gasterophilinae display this red fluorescence emission for a much longer part of the tracheal trunks [21]. Furthermore, the posterior spiracles in species of Gasterophilinae have elongated, slit-shaped openings, whereas species of Hypodermatinae and Oestrinae have spiracular plates with one or two oval or circular openings [1]. Both the sclerotization of the tracheal trunks and the larger posterior spiracular openings likely indicate superior gas exchange capacity in Gasterophilinae. The functional interpretations of these unique morphological features remain speculative and necessitate systematic experimental investigation to elucidate their precise mechanisms and roles.

## 5. Conclusions

This study bridges a significant void in the morphospace characterization of the first instar larva of *C. elephantis*. Furthermore, it corroborates the monophyly of the genus *Cobboldia* and substantiates the phylogenetic relationship of this genus as a sister taxon to *Gasterophilus* + *Gyrostigma* within Gasterophilinae.

## Data Availability

The original contributions presented in this study are included in the article. Further inquiries can be directed to the corresponding author.
